# Light therapy for better mood and insulin sensitivity in patients with major depression and type 2 diabetes: a randomised, double-blind, parallel-arm trial

**DOI:** 10.1186/s12888-015-0543-5

**Published:** 2015-07-24

**Authors:** Annelies Brouwer, Daniël H. van Raalte, Michaela Diamant, Femke Rutters, Eus J.W. van Someren, Frank J. Snoek, Aartjan T.F. Beekman, Marijke A. Bremmer

**Affiliations:** Department of Psychiatry, EMGO Institute for Health and Care Research, VU University Medical Centre and GGZ inGeest, Amsterdam, The Netherlands; Diabetes Centre, VU University Medical Centre, Amsterdam, The Netherlands; Department of Epidemiology and Biostatistics, EMGO Institute for Health and Care Research, VU University Medical Centre, Amsterdam, The Netherlands; Departments of Integrative Neurophysiology and Medical Psychology, Centre for Neurogenomics and Cognitive Research (CNCR), Neuroscience Campus Amsterdam, VU University and Medical Centre, Amsterdam, The Netherlands; Department of Medical Psychology, VU University Medical Centre, Amsterdam, The Netherlands; VU University Medical Centre (ZH4A63), Postal Box 7057, 1007 MB Amsterdam, The Netherlands

## Abstract

**Background:**

Major depression and type 2 diabetes often co-occur. Novel treatment strategies for depression in type 2 diabetes patients are warranted, as depression in type 2 diabetes patients is associated with poor prognosis and treatment results. Major depression and concurrent sleep disorders have been related to disturbances of the biological clock. The biological clock is also involved in regulation of glucose metabolism by modulating peripheral insulin sensitivity. Light therapy has been shown to be an effective antidepressant that ‘resets’ the biological clock. We here describe the protocol of a study that evaluates the hypothesis that light therapy improves mood as well as insulin sensitivity in patients with a major depressive episode and type 2 diabetes.

**Methods/design:**

This study is a randomised, double-blind, parallel-arm trial in 98 participants with type 2 diabetes and a major depressive episode, according to DSM-IV criteria. We will assess whether light therapy improves depressive symptoms and insulin sensitivity, our primary outcome measures, and additionally investigate whether these effects are mediated by restoration of the circadian rhythmicity, as measured by sleep and hypothalamic-pituitary-adrenal axis activity. Participants will be randomly allocated to a bright white-yellowish light condition or dim green light condition. Participants will undergo light therapy for half an hour every morning for 4 weeks at home. At several time points, namely before the start of light therapy, during light therapy, after completion of 4 weeks of light therapy and after 4 weeks follow-up, several psychometrical, psychophysiological and glucometabolic measures will be performed.

**Discussion:**

If light therapy effectively improves mood and insulin sensitivity in type 2 diabetes patients with a major depressive episode, light therapy may be a valuable patient friendly addition to the currently available treatment strategies. Additionally, if our data support the role of restoration of circadian rhythmicity, such an observation may guide further development of chronobiological treatment strategies in this patient population.

**Trial registration:**

The Netherlands Trial Register (NTR) NTR4942. Registered 13 January 2015.

## Background

Major depression is a mood state characterised by depressed mood and loss of interest or pleasure for at least 2 weeks, together with several other symptoms, such as loss of energy, diminished ability to concentrate, changes in appetite or physical activity and sleep disturbances. Up to 20 % of the type 2 diabetes (T2D) patients suffer from major depression [1], where depression appears to be more persistent and recurrent [2–4] and has adverse effects on health behaviour and treatment adherence [5, 6]. Consequently, depression in T2D patients is associated with an increased risk for diabetes complications and mortality [7–16], as well as an economic burden to society [17, 18]. Hence, adequate treatment of depression in T2D patients is required.

Major depression in T2D patients can be treated by psychotherapy or antidepressant drugs. Meta-analytic evidence has shown that antidepressant treatments, both psychological and pharmacological, are only moderately effective in the reduction of depressive symptoms in T2D patients [19, 20]. Glycaemic improvements from anti-depressant therapies have been very modest [20] and some antidepressant drugs have even shown negative metabolic effects, such as weight gain [21] and glucose dysregulation [22]. There clearly is a need for more efficacious treatment strategies of major depression in T2D patients, which improve both psychiatric symptoms and glycaemic control [23].

Light therapy has been shown to be a patient friendly, non-pharmacological antidepressant, which has an early onset of action and a mild adverse-effect profile [24]. Light therapy has been proven successful in patient groups that are traditionally difficult to treat, such as patients with chronic depression and postpartum depression [25–27], and may therefore be effective in the treatment of major depression in T2D patients. Restoration of the circadian rhythmicity, which is often disrupted in depression, has been assumed to be the mechanism of action of light therapy [28]. The biological mechanism behind this chronobiological effect is that light induces the specialised light sensitive retinal ganglion cells to release glutamate in the suprachiasmatic nucleus (SCN) of the brain, which is often called the ‘biological clock’ [29]. The SCN is involved in the regulation of melatonin release, activation of the hypothalamic-pituitary-adrenal (HPA) axis as well as regulation of sleep-wake patterns. A recent clinical study in elderly patients with non-seasonal depression showed that light therapy improved mood, increased upslope evening melatonin levels, attenuated cortisol release and enhanced sleep efficiency [30]. The SCN is also involved in regulation of glucose metabolism [31], which suggests that light therapy may affect glucose metabolism. Two case reports on the effect of light therapy in patients with seasonal depression and insulin dependent diabetes support the idea of effects of light therapy on glucose metabolism [32, 33].

Sleep disturbances, which are indicative of disturbed circadian rhythmicity, often co-occur with major depression [34] and have been associated with hyperglycaemia and increased rates of incident diabetes [35]. In patients with T2D, sleep disorders are associated with a 23 % higher fasting glucose level, a 48 % increment in fasting insulin levels and a decreased insulin sensitivity as indicated by a 82 % increase in homeostatic model assessment of insulin resistance (HOMA-IR) [36, 37]. Two studies using a hyperinsulinaemic-euglycaemic clamp procedure, which is considered the gold standard for the quantification of whole-body insulin sensitivity, observed strong effects of one single night of sleep restriction on insulin sensitivity in healthy volunteers [38] and in patients with type 1 diabetes [39]. Whether the reversed situation, improved insulin sensitivity, can be reached after restoration of the circadian rhythmicity in patients with sleep disturbances has, to the best of our knowledge, not been tested.

**Fig. 1:**
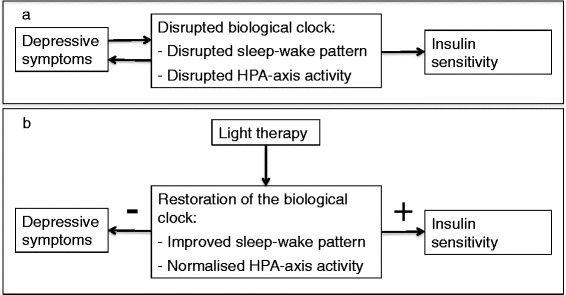
Research hypothesis. Schematic overview of the research hypothesis. **a** Schematic overview of the relationship between the biological clock, depressive symptoms and insulin sensitivity in patients with major depression and type 2 diabetes. **b** Schematic overview of the hypothesised effect of light therapy on the biological clock, depressive symptoms and insulin sensitivity in patients with major depression and type 2 diabetes

We believe that light therapy may be a promising treatment option for patients with major depression and T2D, as light therapy may result in concurrent improvements in depressive symptoms and insulin sensitivity through restoration of the circadian rhythmicity (Fig. 1). To substantiate this theory, we aim to perform a randomised, double-blind, parallel-arm trial that investigates the following hypotheses:

1. Light therapy improves depressive symptoms in patients with a major depressive episode and T2D.
2. Light therapy improves insulin sensitivity in patients with a major depressive episode and T2D.
3. Effects of light therapy on depressive symptoms and insulin sensitivity are mediated by restoration of the circadian rhythmicity, as measured by the sleep-wake pattern and HPA axis activity.

If light therapy proves to be an effective antidepressant in T2D patients and improves insulin sensitivity, light therapy may be a valuable addition to the currently available treatment options for major depression in T2D patients, assuming that improvements in insulin sensitivity may ultimately lead to improvements in glycaemic control.

## Methods/design

### Design

**Fig. 2:**
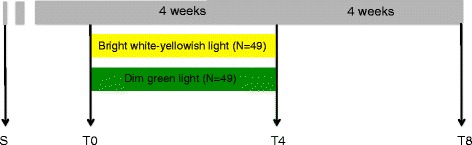
Study protocol. Schematic overview of the study protocol. *Abbreviations*: *S* screening, *T0* start of the intervention, *T4* 4 weeks relative to the start of the intervention, *T8* 8 weeks relative to the start of the intervention

This study entails a randomised, double-blind, parallel-arm trial, consisting of a run-in period of at least 1 week to allow for study effects, a four-week intervention period and a four-week follow-up period. Assessments of primary and secondary outcome measures, as well as mediating factors, will be performed at baseline, during the intervention, after the intervention and after the follow-up period to assess direct and long-term effects of light therapy. The trial methods reporting is consistent with the CONSORT statement [40]. A schematic overview of the trial protocol is provided in Fig. 2.

### Approval

This study will be executed in accordance with the Helsinki Declaration [41]. Participation in the study is voluntary and written informed consent is obtained. Participants can withdraw their consent to participation at any time, without specification of reasons. The ethical committee of the VU University Medical Centre, Amsterdam, The Netherlands, approved this study.

### Participants

Participants will be recruited using a number of strategies: (1) Participants of previous research studies of the VU University Medical Centre will be informed about the study; (2) patients will be informed about the study by affiliated health-care-workers; (3) advertisements in local newspapers, folders and posters in waiting rooms of affiliated health-care-institutions; and (4) via our website. In case patients are interested in participation, a screening consisting of three questions will be applied by telephone. Only patients without a history of laser photocoagulation for the treatment of diabetic retinopathy and scoring 2 or more on one or both questions of the Patient Health Questionnaire-2, a screening tool for a major depressive episode [42], will be invited for a screening visit.

Eligibility for participation will be determined during a screening visit, which constitutes a standardised psychiatric interview, as assessed with the Composite International Diagnostic Interview (CIDI) WHO version 2.1 [43], a medical interview on disease history and medication, physical and blood examination, visual acuity test and fundus photography.

#### Inclusion criteria

Participants will be included when (1) 18 years or older; (2) having a documented physician-based diagnosis of T2D; and (3) having a major depressive episode according to DSM-IV criteria.

#### Exclusion criteria

Participants will be excluded from participation when any of the following criteria is present: (1) a recent history of or current use of light therapy; (2) shift worker; (3) a recent change in antidepressant or blood-glucose lowering medication or therapy (e.g. psychotherapy); (4) use of oral glucocorticoids, melatonin, or cytostatics; (5) pregnancy; (6) psychosis, mania, (probable) dementia, severe drug or alcohol abuse, delirium, and severe acute suicidality; (7) a history of light-induced migraine or epilepsy or severe side effects to light therapy in the past; (8) visual acuity <60 %, diabetic retinopathy EURODIAB grades 3, 4 or 5 (severe non-proliferative or preproliferative retinopathy, photocoagulated retinopathy, proliferative retinopathy) [44, 45], senile macula degeneration; (9) other medical condition or recent medical event that potentially compromises the effects or safety of light therapy.

There are no restrictions with respect to type of antidepressant or blood-glucose lowering medication.

### Safety

Light therapy has proven to be a patient friendly and safe intervention, with little side effects that normally resolve after discontinuation of the therapy.

**Fig. 3:**
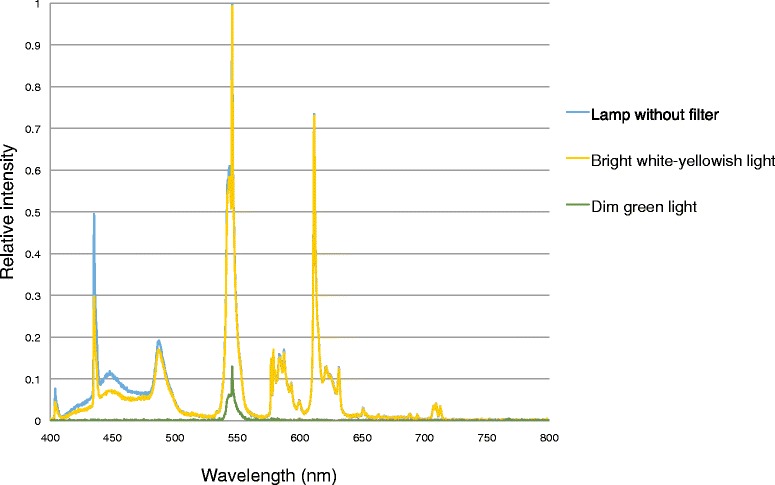
Light spectral and relative intensity measures. Light spectral and relative intensity measures at 50 cm distance of the bright white-yellowish light condition (yellow), the dim green light condition (green) and the lamp without filter (blue)

The ocular safety of light therapy, however, remains matter of debate. Follow-up of patients with cumulative exposure durations up to 1250 h demonstrated that light therapy is safe in patients without pre-existing ocular abnormalities [46]. Ocular effects of light therapy in patients with pre-existing ocular abnormalities, diabetes, or in patients using photosensitising medication, however, remain under speculation [47]. Therefore, stringent ocular exclusion criteria have been defined (e.g. diabetic retinopathy EURODIAB grade 3, 4 and 5) and safety precautions have been taken, such as the use of filters that eliminate an additional part of the energetic light spectrum below 450 nm (blue light), which theoretically has the most damaging potential, in addition to the of UV-A and UV-B filters with which the light therapy lamps are normally equipped with (Fig. 3).

Mania (in bipolar depressive patients) and acute suicidality have been theorised as potential psychiatric side effects of light therapy [24, 48, 49] although there is little documented evidence. The investigator will contact a participant weekly during the intervention in order to be able to intervene in case of deterioration of psychiatric symptoms.

### Intervention

The intervention will consist of light therapy for 30 min every morning, for four consecutive weeks at home. The time at which light therapy is applied in the morning will be determined according to results from the Morningness-Eveningness Questionnaire [24] to allow for accurate phase timing, but in agreement with the participant, to make sure the light therapy can be incorporated in the participant’s daily rhythm. A timer will be installed to provide a 90 min time window in which the participants can follow the 30 min of light therapy. This provides the participants with a time window of an hour to start the light therapy, which will prevent participants from taking light therapy at another time of the day. Participants will sit in front of the light boxes at a distance of 50 cm and at an angle of 45 °, while they eat breakfast or do some reading (protocol as recommended by Wirz-Justice and colleagues, 2009 [50]).

Diamond-5 SAD lightbox (CE certified; SAD Lightbox Company, Berkshire, United Kingdom) light therapy lamps will be used. The lamps will be equipped with heat-resistant filters, to create an experimental and placebo light therapy condition.

The experimental condition will be bright white-yellowish light, with an intensity of 10.000 lx at a distance of 50 cm. The lamps will be equipped with high-throughput pale yellow filters (LCT Yellow, Model 212; Lee Filters, Burbank, California, United States of America), to filter the spectrum below 450 nm for ocular safety. The circadian system appears most sensitive to light with short wavelengths and light therapy with light of around 470 nm is able to induce a substantial phase advance [51]. We therefore think filtering for ocular safety will not interfere with our hypothesis. Spectral and intensity measures are depicted in Fig. 3.

The control condition will be dim green light with an intensity of 450 lx at a distance of 50 cm. As the green part of the spectrum around 545 nm is biologically relatively inactive [51], we expect there to be no substantial effects, especially at this low intensity, although expectations will be quite comparable to white light, as was previously shown for dim red light [52]. To create the dim green light condition the lamps will be equipped with low-throughput dark green filters (Aurora Borealis Green: Model 740; Lee Filters, Burbank, California, United States of America). Spectral and intensity measures are depicted in Fig. 3.

### Compliance

Participants will be instructed by research assistants on how to follow the light therapy program. The position of the light therapy lamp and the participant during light therapy will be marked, so the participant can retake the same position day after day. The investigator will contact the participants weekly during the intervention period and compliance will be monitored and reinforced when needed.

### Randomisation

Participants will be randomly assigned to the experimental or placebo condition at a 1:1 ratio. Randomisation will be performed in blocks of 6 using a computer-generated table and will be stratified for whether a participant will or will not take part in the hyperinsulinaemic-euglycaemic clamp experiment, which will only be performed in a subgroup of the participants.

### Blinding

Both investigators and participants will be blind with respect to the treatment conditions. Participants will be informed that two different colours of light (white-yellowish and green) will be compared, but not that a placebo condition is present, nor that the green light is less bright or less effective compared to the white-yellowish lamp. This approach is effective in avoiding differences in participants’ expectations regarding the effectiveness of the conditions, as expectations may affect treatment effects [30, 53, 54]. The investigators involved in data acquisition will be blind to the conditions, as the lamps will be delivered at the participants’ homes by independent research assistants, who are instructed not to discuss the brightness of the lamp, and participants will be instructed not refer any details of their condition to the investigators. A similar blinding procedure showed to be successful in a previous study conducted by our institute [30].

To evaluate the blinding procedure, participants’ expectations of the effect of light therapy on depressive symptoms will be measured before installation of the light therapy lamps, using an adapted version of the expectations questionnaire developed by Lieverse and colleagues, 2011 [30].

### Study setting

Test visits will be carried out at clinical research units of two locations of the VU University Medical Centre: Amsterdam and Hoorn, The Netherlands.

### Outcome measures

Outcome measures will be evaluated just before the start of the intervention (T0), after 4 weeks of light therapy (T4) and after 4 weeks of follow-up (T8). Changes in outcome measures (T0 – T4, T4 – T8, or T0 – T8) will be compared between the two study conditions. Additionally, several outcome measures will also be evaluated weekly during the intervention period (T0, T1, T2, T3, T4, T8) to allow for analysis or temporal changes in these measures. A schematic overview of the outcome measures is depicted in Table 1.

**Table 1:** Outcome measures

Weeks relative to start intervention (T)			0	1	2	3	4	5	6	7	8
Visits		S	1				2				3
Telephone contact				1	2	3			4		
Run-in phase		X-es	X-es								
Intervention phase				X-es	X-es	X-es	X-es				
Follow-up phase								X-es	X-es	X-es	X-es
Participant characteristics	Psychiatric diagnosis, seasonality, chronotype, sleep apnoea, medical history, current medication, biochemical blood tests, sociodemographic factors	X	X								
Psychometrical measures	Depressive symptoms		X	X	X	X	X				X
	Anxiety symptoms		X	X	X	X	X				X
	Diabetes-related distress		X				X				X
	Quality of life, disability		X				X				X
Glucometabolic measures	Insulin sensitivity		X				X				
	HbA1c level		X				X				X
	Fasting glucose level		X				X				X
	Hypoglycaemic events		X	X	X	X	X				X
Circadian mediating factors	Diurnal HPA axis activity		X				X				X
	Objective sleep		X				X				X
	Subjective sleep		X	X	X	X	X				X
Other factors	Body weight, body mass index, waist-hip ratio, body composition		X				X				X
	Physical activity		X				X				X
	Autonomic nervous system function		X				X				X
Safety	Adverse effects		X	X	X	X	X				X
	Ophthalmological examination	X									X
Compliance	Participant-report compliance			X	X	X	X				
	Light meter		X				X				X

*Abbreviations*: *S* screening, *HPA* hypothalamic-pituitary-adrenal

#### Participant characteristics

Various participant characteristics will be obtained before the start of the light therapy (S, T0): psychiatric diagnosis in the category of mood and anxiety disorders according to DSM-IV criteria as assessed using the CIDI WHO version 2.1 [43]; seasonality of depression [55]; chronotype [24]; presence of sleep apnoea [56]; medical history; current medication; biochemical blood tests; and sociodemographic factors. These participant characteristics allow for the characterisation of our study sample.

#### Primary outcome measures

Depressive symptoms will be assessed by the self-report version of the Inventory of Depressive Symptomatology (IDS) (T0, T1, T2, T3, T4, T8) [57]. The IDS allows for profiling of depression, as the questionnaire discriminates directions of the weight, appetite, sleep and psychomotor symptoms, and can be used to determine depression severity. Scores between 14 and 25 indicate mild depression; scores between 26 and 38 indicate moderate depression; scores between 39 and 48 indicate moderate to severe depression; and scores of 49 and greater indicate severe depression. A reduction of 50 % or greater of the baseline depression score is regarded as a clinically significant reduction in depressive symptoms. Depression remission is *a priori* defined as an IDS score of 13 or less.

Insulin sensitivity will be evaluated at the baseline visit (T0) and the visit following light therapy (T4) in a subsample of 60 participants. Insulin sensitivity will be quantified using a hyperinsulinaemic-euglycaemic clamp procedure, the gold standard for the quantification of whole-body insulin sensitivity. This procedure quantifies the rate of exogenous glucose infusion required to maintain the plasma glucose concentration at euglycaemic levels in response to a fixed increase in the plasma insulin level. Because 80–90 % of the infused glucose is taken up by skeletal muscle under conditions of euglycaemic hyperinsulinaemia, insulin sensitivity measured with this procedure primarily reflects that of skeletal muscle [58]. The hyperinsulinaemic-euglycaemic clamp procedure is performed as previously described by DeFronzo and colleagues [59]. Briefly, participants will be admitted at our research unit following an overnight fast. They are placed in a semi recumbent position during the procedure. A cannula will be inserted into a vein in the antecubital fossa for infusion of glucose and insulin. Insulin (Novorapid 100 IU/ml; Novo Nordisk, Bagsvaerd, Denmark) will be continuously infused at a rate of 40 mU/m2*min, which creates a hyperinsulinaemic state, which will largely decrease endogenous glucose production by the liver. A contralateral dorsal hand or wrist vein will be cannulated and maintained at 50 °C to permit sampling of arterialised venous blood for bedside measurement of blood glucose concentrations (Yellow Springs Instrument 2300 STAT Analyser, Yellow Springs, Ohio, Unites States of America) every 5 min. Glucose (20 %) will be infused at a variable rate to maintain blood glucose levels at 5 mmol/L. When a stable blood glucose level of 5 mmol/L is achieved (minimally after 90 min of insulin infusion), the blood glucose level will be kept at this level for 30 min (steady state). At this point the glucose infusion rate equals the skeletal muscle glucose uptake. The M-value (expressed as the amount of glucose infusion in mol/kg*min) will be calculated over this steady state period as described previously [59]. Blood samples for measurement of plasma insulin concentrations will be obtained at several time points before and during insulin infusion.

#### Secondary outcome measures

Secondary participant-reported outcome measures include anxiety symptoms, as anxiety symptoms often co-occur with depressive symptoms; diabetes-related distress; and quality of life and disability, as these measures are increasingly important when evaluating new treatments in clinical trials. Anxiety symptoms are evaluated using the Beck Anxiety Inventory (T0, T1, T2, T3, T4, T8) [60], diabetes-related distress is measured using the Problem Areas In Diabetes questionnaire (T0, T4, T8) [61], quality of life using the EuroQol-5 Dimensions questionnaire (T0, T4, T8) [62] and disability using the Sheehan Disability Scale (T0, T4, T8) [63].

Glucometabolic secondary outcome measures will include the clinically relevant glycated haemoglobin A1c (HbA1c) level (T0, T4, T8), fasting plasma glucose level (T0, T4, T8), and number of hypoglycaemic events as reported in the diary (T0, T1, T2, T3, T4, T8).

#### Circadian mediating factors

Mediating factors that reflect circadian rhythmicity will include diurnal HPA axis activity, objective accelerometer measures of sleep duration and subjective measures of sleep duration and quality.

Diurnal HPA-axis activity is assessed by free cortisol assessment using six saliva samples per day (T0, T4, T8). The morning cortisol awakening responses will be assessed by four saliva samples in the morning: after awakening, plus 30 min, plus 45 min and plus 60 min. Cortisol levels in the evening will be obtained from two saliva samples in the evening at 10 and 11 pm. Saliva samples will be collected at home, using cotton swaps (Salivette, Sarstedt, Nümbrecht, Germany) that will be stored in the refrigerator. Participants will bring the samples with them to the research facility where the saliva will be centrifuged and stored at −80 °C for analysis.

Objective measures of sleep will be obtained using a microelectromechanical system (GENEActiv, Activeinsights Ltd, Kimbolton, United Kingdom), an accelerometer that can reliably measure sleep-wake behaviour [64] and will be worn for one week continuously. Estimates of sleep parameters will be obtained from these data using a previously validated algorithm [64].

Subjective sleep parameters will be scored using the Pittsburg Sleep Quality Index (T0, T4, T8) [65], the Insomnia Severity Index (T0, T2, T4, T8) [66, 67] and a sleep diary (T0, T1, T2, T3, T4, T8) [68].

#### Other factors

Other potential mediating or confounding factors include body weight, body mass index, waist-hip ratio, body composition, physical activity and autonomic nervous system function. Body composition will be measured by bioelectrical impedance analysis (T0, T4, T8), physical activity will be measured using a wrist-worn accelerometer (T0, T4, T8) and autonomic nervous system function will be evaluated with spectral analysis of the heart rate variability (T0, T4, T8). Whole blood (T0, T4), plasma and serum (T0, T4, T8) will be collected and stored for future additional research questions that require additional biochemical or DNA analyses.

#### Safety and tolerability of light therapy

Adverse effects of light therapy will be assessed, using a self-report adverse effects questionnaire (T0, T1, T2, T3, T4, T8) that is based on the side effect interview from Lieverse and colleagues, 2011 [30, 69]. The following items will be assessed: tired eyes, dry eyes, painful eyes, visual impairment, headache, early morning awakening, fatigue, drowsiness, weakness, nervousness, anxiety, agitation, irritability, dizziness, nasal congestion, fever, seating, hypoglycaemia, hot flushes, allergy, rash, cough, dry mouth, nausea, vomiting, decreased appetite, increased appetite, constipation and diarrhoea. Presence and severity of each item will be rated as absent, mild, moderate or severe.

Additionally, we will assess ophthalmological effects, by measures of visual acuity, contrast sensitivity and fundus photography. An ophthalmologist will perform grading of the photographs according to the EURODIAB study grading system [5, 44, 45].

#### Compliance

Participants will note their compliance to therapy in a diary. Objective measures of compliance will be obtained from lux-meters built in a wrist-worn accelerometer.

### Sample size

A previous study on the effects of light therapy on depression in elderly patients using a comparable protocol [30] showed that direct treatment effects of light therapy on depressive symptoms were moderate (effect size 0.50 Cohen’s d), and increased during follow-up (effect size 0.91 Cohen’s d), with one-sided testing. Power calculations were based on the expected moderate effect size and a conservative repeated measures correlation of 0.6, using conventional values for α (0.05) and β (0.20), and two-tailed tests with equal groups – the sample size aimed for will be 42 participants per treatment arm. Accounting for an attrition rate of 15 %, we derive at a total sample size of 98 participants.

The question whether light therapy alters insulin sensitivity in type 2 diabetes patients with depression will be tested in a subsample of 60 participants in total. A sample size of 30 participants per treatment arm is sufficient to detect a moderate effect (0.76 Cohen’s d) of light therapy on insulin sensitivity, with conventional values for α (0.05) and β (0.20), and two-tailed tests with equal groups, assuming a dropout rate of 10 %. The hyperinsulinaemic-euglycaemic clamp experiment will thus be performed in a subgroup of the participants and participation in this procedure will be optional. Participants with difficult venous access or medical conditions that impair the safety of the procedure will be excluded from participation in the hyperinsulinaemic-euglycaemic clamp experiment.

### Statistical analyses

Data will be analysed using SPSS (version to be determined). Participant and baseline characteristics across treatment groups will be compared using analysis of variance (ANOVA) and chi-squared statistics. Analyses of our primary outcome measures will be performed on the basis of intention to treat. The superiority of the bright white-yellowish light condition compared to dim green light condition will be tested using a repeated measures mixed models design. Both treatment (independent of time) and condition*time interaction effects will be evaluated. Repeated measures logistic regression will be applied to test differences in depression remission rates. Preacher and Hayes mediation analysis will be performed to test whether the effects of light therapy are mediated by restoration of the circadian rhythmicity. Post hoc analyses of this study may include subgroup or discriminant analysis to test if participant characteristics can be identified that predict positive effects of light therapy, such as baseline depression severity, baseline depressive symptoms profile, baseline insulin sensitivity, baseline HbA1c, chronotype, or baseline insomnia severity, seasonality of depression and time of year at which the participant was included in the study.

## Discussion

To the best of our knowledge, we are the first to investigate the effects of light therapy on depressive symptoms and insulin sensitivity in T2D patients with a major depressive episode.

Creating a valid placebo condition is a major challenge in light therapy trials, due to the visibility of light. In previous research various placebo conditions have been used: deactivated air ionisation generators, dim red light placebo conditions, dim white light conditions and inappropriate timing of light therapy [30]. We however decided to use a dim green light condition as placebo for several reasons. Firstly, inappropriate timing of light therapy can be difficult, as phase alterations vary in non-seasonal depression. Secondly, we regard a dim white light condition less credible when compared to a coloured dim light condition, as a coloured glare to the surroundings is absent for a white light condition. Thirdly, a dim red light placebo condition may be less credible nowadays, as much more information on (e.g. on the active spectrum of light) has become available to the public. We considered dim green light a valid placebo condition, reasoning that because the green part of the spectrum around 545 nm is biologically relatively inactive [51], at low intensities there will be no substantial effect, while dim green light will induce positive expectations quite comparable to white light, as was previously shown for dim red light [52].

Participation in the hyperinsulinaemic-euglycaemic clamp experiment is optional, to prevent participants to refrain from participation in our study. This procedure may introduce a selection bias, with potentially psychiatrically and somatically less severely affected participants in the hyperinsulinaemic-euglycaemic clamp subgroup, limiting the generalizability of our findings on insulin sensitivity.

Participants with diabetic retinopathy EURODIAB grade 3–5 will be excluded from participation in this study as a safety precaution. If our study does find positive effects on mood and insulin sensitivity in absence of ocular damage, it may be worthwhile to investigate the effects of light therapy in patients with diabetic retinopathy EURODIAB grade 3–5, as these patients are expected to have a greater risk for depression and poor glycaemic control.

## Conclusions

If light therapy shows to be safe and effective in the reduction of depressive symptoms and improves insulin sensitivity, light therapy may be a valuable patient friendly addition to the currently available treatment strategies for major depression in T2D patients, assuming that improved insulin sensitivity may lead to improvements in glycaemic control, as measured by fasting glucose levels and HbA1c levels. Additionally, if our data support the role of restoration of circadian rhythmicity in these effects, such a finding may guide further development of chronobiological treatment strategies in these patients, as well as provide a basis for research into the effects of light therapy in other T2D patients, such as those without major depression but with sleep disorders.

## Trial status

The first participant was enrolled in June 2014. At the time of submission of this study protocol participants were still being recruited.

## Abbreviations

IDS: Inventory of depressive symptomatology
CIDI: Composite International Diagnostic Interview
HbA1c: glycated haemoglobin A1c
HPA: Hypothalamic-pituitary-adrenal
SCN: Suprachiasmatic nucleus
T2D: Type 2 diabetes
